# Use of a special Brazilian red-light emitting railroad worm Luciferase in bioassays of NEK7 protein Kinase and Creatine Kinase

**DOI:** 10.1186/s12858-017-0087-z

**Published:** 2017-07-19

**Authors:** Arina Marina Perez, Bruno Aquino, Vadim Viviani, Jörg Kobarg

**Affiliations:** 1 Instituto de Biologia, Departamento Bioquímica e Biologia Tecidual, Universidade Estadual de Campinas, Campinas, Programa de Pós-gradução em Biologia Molecular e Funcional São Paulo, Rua Monteiro Lobato 255, Campinas, SP CEP 13083-862 Brazil; 2 Faculdade de Ciências Farmacêuticas, Universidade Estadual de Campinas, Campinas, São Paulo Brazil; 3 Laboratório Nacional de Biociências, Centro Nacional de Pesquisa em Energia e Materiais, Campinas, São Paulo Brazil; 4 Laboratório Bioquímica e Biotecnologia de Sistemas Bioluminescentes, Departamento Física, Química e Matemática, CCTS, UFSCAR, Sorocaba, Brazil

## Abstract

**Background:**

Luciferases, enzymes that catalyze bioluminescent reactions in different organisms, have been extensively used for bioanalytical purposes. The most well studied bioluminescent system is that of firefly and other beetles, which depends on a luciferase, a benzothiazolic luciferin and ATP, and it is being widely used as a bioanalytical reagent to quantify ATP. Protein kinases are proteins that modify other proteins by transferring phosphate groups from a nucleoside triphosphate, usually ATP.

**Methods:**

Here, we used a red-light emitting luciferase from *Phrixotrix hirtus *railroad worm to determine the activity of kinases in a coupled assay, based on luminescence that is generated when luciferase is in the presence of its substrate, the luciferin, and ATP.

**Results:**

In this work we used, after several optimization reactions, creatine kinase isoforms as well as ﻿NEK7 protein kinase in the absence or presence of ATP analogous inhibitors  to validate this new luminescence method.

**Conclusion:**

With this new approach we validated a luminescence method to quantify kinase activity, with different substrates and inhibition screening tests, using a novel red-light emitting luciferase as a reporter enzyme.

**Electronic supplementary material:**

The online version of this article (doi:10.1186/s12858-017-0087-z) contains supplementary material, which is available to authorized users.

## Background

Luciferases from bioluminescent organisms have many applications in biomedical areas, ranging from bioengineering, biosensors, cell biology to competition assays for bioassay development.

One of the most important sources of luciferases are the bioluminescent beetles belonging to the Elateroidea superfamily, within Lampyridae (fireflies), Phengodidae (railroadworms) and Elateridae (click-beetles) [1, 2]. They emit different bioluminescence colors depending on life stage and the lantern type, from green to red, that serve as ecological adaptations to different photic environments and for diverse biological functions.

The firefly luciferin-luciferase system is one of the best studied ones. Beetle luciferases are bi-functional enzymes with CoA-ligase and oxygenase activities [1]. The firefly luciferases consist of a monomer of approximately 60 kDa and 550 amino acids residues [3]. They catalyze the bioluminescent reaction involving firefly luciferin (D-LH2: a benzothiazolic compound), adenosine triphosphate (ATP), magnesium ion and molecular oxygen with the formation of an electronically excited species (oxyluciferin), inorganic pyrophosphate (PPi), carbon dioxide and adenosine monophosphate (AMP) [1].

Firefly luciferase catalyzed reaction:

$$ \mathrm{Luciferase}+\mathrm{ATP}+\mathrm{luciferin}+{\mathrm{O}}_2\to \mathrm{AMP}+\mathrm{PPi}+\mathrm{Oxyluciferin}+{\mathrm{CO}}_2+\mathrm{light} $$

This reaction follows the Michaelis– Menten equation for both ATP and luciferin substrates and can be used to quantitatively measure the ATP reaction.

The firefly luciferin-luciferase reaction became widely used for analytical purposes because light can be easily detected with high sensitivity and with simple instrumentation. Furthermore, light emission can be measured in different types of vessels, even inside cells, living animals or plants, because ATP provides the activation energy for all living cells and is used as cofactor by hundreds of enzymes.

Throughout evolution, cells have adapted the K_M_ values of most of their enzymes to allow efficient regulation of their enzyme activities. Consequently, most living cells have similar intracellular ATP concentrations. The quantity of this molecule per cell is therefore mainly determined by the intracellular volume: normal bacterial cells contain 1–2 attomoles ATP, whereas larger mammalian cells typically contain 10,000–100,000 attomoles [4].

Due to their high bioluminescence efficiency (15–61%), beetle luciferases, especially firefly luciferases and their genes, have been extensively used in analytical, pharmaceutical, biomedical, and diagnostic applications, and as reporter genes in molecular biology. High-throughput screening, determination of ATP, microbial detection, immunoassays, nucleic acid assays, nucleotide analysis, biosensing and bioimaging are some of the common applications of luciferase [5–10]. However, most assays use firefly luciferases that emit in the yellow-green region and are pH-sensitive, thereby in some cases limiting the applicability to samples**.** Thus the cloning of South-American *Phrixotrix* rail roadworm red light luciferase increased the range of applications, extending it to usage in cultured mammalian cells, which are regularly grown in culture medium that contains phenol red, a compound that absorbs light in the yellow/green parts of the spectrum.

Among beetle luciferases, the red-emitting luciferase of *Phrixotrix hirtus* railroad worm, previously cloned and characterized by Viviani et al. [6] is the only luciferase which naturally emits red bioluminescence, with the most red-shifted and narrowest emission spectrum (623 nm). Furthermore, this luciferase displays one of the lowest K_M_ values for luciferin [7, 8, 10]. These properties make this enzyme especially suitable for ATP assays in pigmented samples. The aim of this paper was to develop a simple, low cost, “all in-one-reaction tube”, competition assay to evaluate substrates and inhibitors for a broad spectrum of kinases, represented here by the NEK7 protein kinase and the creatine kinase, using *P. hirtus* red light emitting luciferase.

## Methods

### Cloning and recombinant protein expression

#### Red emitting luciferase expression

The gene that encodes red light emitting luciferase was originally cloned from *P. hirtus* railroad worm luciferase [6], sub-cloned in the pCold vector [3, 6] and provided by one of the authors (V.V.). The luciferase was expressed in *E. coli* BL21 cells in presence of 0.3 mM of isopropyl-β-D-thio-galactoside (IPTG), and purified according established procedures. After expression, the 6xHis-red luciferase fusion protein was purified using a Hitrap chelating HP column. The column and the lysate were equilibrated in Buffer A (20 mM sodium phosphate pH 8.0; 0.5 M NaCl; 20 mM imidazole) and eluted in Buffer B (20 mM sodium phosphate pH 8.0, 0.5 M NaCl, 500 mM imidazole). The eluate containing red luciferase was dialyzed to Tris-HCl buffer (pH 8.0). After purification the luciferase preparation was tested for the absence of kinase or other ATP consuming enzyme activities, which may have been co-purified from *E. coli* lysate (Additional file 1: Figure S1).

#### Creatine Kinase expression

Genes coding for 6xHis-brain (CKB) and 6xHis-muscle (CKM) creatine kinase were subcloned from pEF1α [11] with *Eco*RI/*Not*I into plasmid pET28a. Plasmids containing 6xHis-sarcomeric (pUS01) and 6xHis-ubiquitous (pUS04) cloned creatine kinase in pET3b vector were kindly provided by Dr. Schlattner [12]. All four plasmids were transformed in *E. coli* BL21(DE3) (Novagen) and protein expression was initiated by adding 0.5 mM of IPTG followed by incubation for 16 h at 30 °C. Cells were harvested and ressuspended in Buffer CK-A (50 mM Tris-HCl, pH 7.4; 250 mM NaCl; 20 mM Imidazole; 50 μg/ml lysozyme) for 1 h, prior sonication. Lysates were centrifuged at 20.000 x g for 40 min at 4 °C and proteins were purified using a HiTrap Chelating HP column. Elution was performed using a gradient of imidazole. Purified proteins were dialyzed against Buffer CK-A without imidazole and frozen at −80 °C until usage.

#### NEK7, Mat1, NEK9 and CC2D1A expression

The GST-tagged protein fragments/domains, as retrieved from yeast-two hybrid screen [13]:NEK9(764–976), CDK-activating kinase assembly factor MAT1(Full-length) and CC2D1A(501–940) were expressed in *E. coli* BL21 (DE3) cells using 1 mM IPTG, at 25 or 30 °C, respectively, for 4 h. The full-length human NEK7 protein (6 × His-NEK7) was expressed in *E. coli* BL21 (DE3) cells, as described previously [13]. Expression was induced for 4 h using 1 mM IPTG at 28 °C. Induced cells were harvested and lysed by sonication in extraction buffer (50 mM HEPES, pH 7.5; 5 mM sodium phosphate, 300 mM NaCl, 5% glycerol, 1 mM PMSF, 625 μg/mL lysozyme). The cell lysates were clarified by centrifugation with 16.000×g for 10 min at 4 °C in order to obtain the supernatant. Cleared fractions were purified by GST affinity chromatography using a Glutathione Sepharose 4 Fast Flow resin. GST fusion proteins were eluted in buffer containing 50 mM Tris-HCl and 50 mM reduced glutathione at pH 8.0. Cleared lysates containing 6 × His-NEK7, were purified by affinity liquid chromatography, using a HiTrap Chelating Affinity Chromatography Column (GE Healthcare), followed by elution with a linear concentration gradient of imidazole (1 to 100 mM) in extraction buffer. All of the eluted recombinant proteins were dialyzed against kinase buffer (50 mM MOPS, pH 7.5, 300 mM NaCl, 10 mM MgCl_2_, 0.1 mM PMSF).

### Luciferase assay optimization

Luciferase activity assays were performed using an Enspire multimode plate reader (Perkin Elmer) through luminescence measurement mode. As initial conditions we employed 1 μM luciferase, 100 μM luciferin (Sigma, L9504) and 1 μM ATP.

#### ATP titration

The reaction contained: 50 mM Tris-HCl pH 8.0; 4 mM MgSO_4_; 1 μM luciferase; 100 μM D-Luciferin and different ATP concentrations (0 nM; 50 nM; 500 nM; 1 μM; 50 μM; 500 μM; 1 mM; 2 mM).

#### pH titration

Reaction contained: 4 mM MgSO_4_; 1 μM luciferase; 100 μM luciferin; 1 μM ATP and 50 mM Tris-HCl at different pHs (9.0; 8.0; 7.0; 6.0).

#### Luciferin titration

To determine the ideal concentration of luciferin, we set up the reactions with: 50 mM Tris-HCl, pH 8.0; 4 mM MgSO_4_; 1 μM luciferase, 1 μM ATP and different concentrations of D-luciferin (0 μM; 0.5 μM; 50 μM; 100 μM; 500 μM; 1 mM; 2 mM; 4 mM).

#### ATP consumption assay

The activities of two kinases (creatine kinase and NEK7) were measured using luciferase. The reaction for creatine kinase (1 μM) contained 50 mM Tris-HCl, pH 8; 4 mM MgSO_4_; 50 μM ATP and 5 mM creatine as a phosphate receptor. For the NEK7 assay on the other hand we used 50 mM Tris-HCl, pH 8; 4 mM MgSO_4_; 1 μM ATP and 0.5 mM of NEK7 protein and of its known, previously characterized, protein substrates/protein interactors [13]. These reactions were incubated at 37 °C for 30 min and the ATP consumption was measured by adding 100 μM luciferin and 1 μM luciferase followed by immediately reading of the emitted luminescence.

#### Inhibition assay

In order to identify new inhibitors of NEK7 kinase we used the same standard reaction adding 100 μM of each inhibitor. All reactions contained 100 μM luciferin, 1 μM luciferase, 1 μM ATP in assay buffer (50 mM Tris-HCl, pH 8; 4 mM MgSO_4_) in absence or presence of 0.5 μM protein substrate (NEK9). The inhibitors were added in DMSO. In controls (columns 1–3), we added the same final concentration of DMSO (0.8%).

## Results

### Luciferase assay optimization

**Fig. 1:**
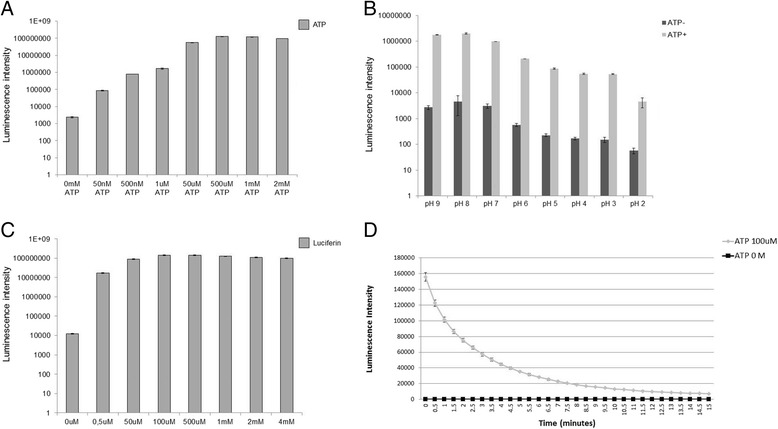
Luciferase assay optimization. **a** ATP titration: The graph represents the light intensity of each reaction containing different ATP concentrations as indicated. **b** pH titration: The graph represents the light intensity of each reaction at different pH. **c** Luciferin titration: The graph represents the light intensity of each reaction using different luciferase concentrations as indicated. Graphs A, B and C are in logarithm scale. **d** Light emission decay time: The graph represents the time that the red luciferase emits light. The means and standard deviations of all graphs were obtained from quadruplicate experiments

To start luciferase activity assay optimization, we first expressed and purified red luciferase protein (Additional file 1: Figure S2A). After this we determined the ideal ATP concentration and pH for reaction (Fig. 1a and b), as well as the luciferin concentration (Fig. 1c) as described in methods section. As shown in in the graph in Fig. 1a, concentrations above 500 μM of ATP saturated the reaction. Based on this, we choose 1 μM ATP for all assay because it is a concentration that does not saturate the reaction, except to CK consumption assays where we used 50 μM ATP to improve the signals in the reaction.

In relation to pH titration, as shown in Fig. 1b the maximum activity of luciferase was obtained at pH 8.0, as previously reported [6, 7]. However, it still shows high intense luminescence in the whole range from pH 9 to 7, indicating that this luciferase can be used in experiments using different pHs.

Next, we tested the luciferin concentration and found that concentrations above 100 μM were saturating (Fig. 1c). Since we want to focus on measuring ATP, the substrate luciferin should not be at limiting levels and on the other hand the cost factor is also relevant. Therefore, we used 100 μM of luciferin in the other assays (Fig. 1c).

Since the emission of light by luciferase is very fast and follows a flash-like kinetics, we also analyzed the time of decay of light emission. As shown in Fig. 1d, we verified that the decay of light emission is very fast and that after 15 min no more light emission was detectable. Therefore, the measurement of the ATP concentration must be performed immediately without any pre-incubation period.

The purity of luciferase used in this method must be very high, because any contamination by proteins that consume ATP will give false positive background result. To test if there was any contamination of this kind we pre-incubated the purified luciferase for 30 min with ATP but without luciferin and compared the intensity of emitted luminescence with a reaction without previous incubation (Additional file 1: Figure S1). Had there been a contamination, the intensity of luminescence would be lower in the condition with a 30 min pre-incubation. However, both test conditions resulted in very similar luminescence intensities, thereby indicating that the purified luciferase was not contaminated with ATP-consuming proteins (see also Additional file 1: Figure S2A for purity of protein samples).

Together, these experiments demonstrate optimization of the assay conditions for red luciferase-based detection of ATP, for screening substrates and inhibitors of kinases.

### Applications: A luciferase assay to detect kinase activity and to screen for kinase inhibitors

Railroad worm red-emitting luciferase, similar to firefly luciferases, emits light dependent on ATP, magnesium and luciferin. This assay is based on luminescence intensity, where the luminescence produced is directly proportional to the ATP concentration. Therefore, this assay can be used to measure the activity of any protein that consumes or produces ATP. To test this, we employed two kinases. One is the kinase NEK7, that modifies other proteins by covalently adding phosphate groups to them (phosphorylation), and the other one is creatine kinase (CK), which can transfer a phosphate group from ATP to creatine, to form phosphocreatine.

**Fig. 2:**
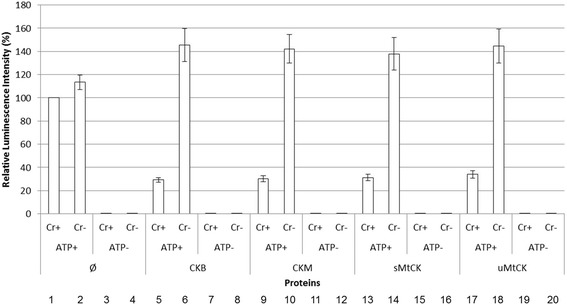
ATP consumption by creatine kinases. 1 μM of each of the four purified isoforms of creatine kinase were incubated for 30 min with (ATP+/Cr+) or without (ATP−/Cr-) 50 μM of ATP and 5 mM of creatine. Luminescence was measured using red-luciferase and normalized by positive control (without creatine kinase, with 50 μM of ATP and 5 mM creatine). Standard deviation represent three independent experiments. Ø – without creatine kinase; CKB – brain creatine kinase; CKM – muscle creatine kinase; uMtCK – ubiquitous mitochondrial creatine kinase; sMtCK – sarcomeric mitochondrial creatine kinase

CK has four isoforms (uMtCK, sMtCK, CKB and CKM) and all of them were tested for ATP consumption. In Fig. 2 we can observe that in the absence of any kinase there is no ATP consumption and hence no decrease in luciferase light emission, even in the presence of creatine (columns 1 and 2).

In the absence of ATP, there is no luminescence emission, since luciferase requires ATP for bioluminescence (columns 3, 4; and also 7, 8, 11, 12, 15, 16, 19, 20). When we incubated CK protein isoforms with ATP but without creatine there was no decrease in light emission, indicating no variation in ATP concentration (columns 6, 10, 14, 18). However, when creatine was added, the light emission was significantly decreased to 20–35% of the initial values (compare columns 5, 9, 13, 17 to column 1). These results show that all CKs were active. Therefore, in principle this method can be efficiently used to measure ATP consumption by all four CK isoforms through a decrease of light emission in the coupled red luciferase assay.

The other kinase used to test this method was the serine/threonine protein kinase NEK7, which has roles in mitotic spindle formation, cytokinesis, and centrosome duplication, and which has been found over-expressed in breast, colorectal, laryngeal and lung cancer as well as in non-Hodgkin lymphoma. To identify substrates of NEK7 kinase [13] and to identify inhibitors, we previously performed screening assays with commercial kinase assays and kinase inhibitor libraries [14]. Here, we were interested to develop a sensitive but less costly assay, using red-emitting luciferase as a reporter enzyme in competitor methods according to the methods section above.

**Fig. 3:**
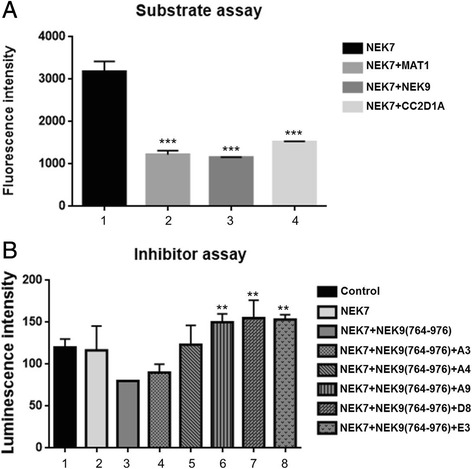
Luciferase assay as competitor in substrates and inhibitor screening assay. **a** NEK7 substrates testing through the competition assay luciferase activity. Graph represents the intensity of luminescence reactions were performed with NEK7 in the presence or absence of different protein substrates (NEK9 (764–976), Mat1(Full-length), CC2D1A (501–940) as indicated in the legend. (***) Indicates *P* < 0·05 calculated using one-Way ANOVA, Multiple comparison test (GraphPad Prism). **b** Test of NEK7 inhibitors by competition assay using red luciferase. All conditions contain 100 μM Luciferin, 1 μM luciferase, 1 μM ATP in Buffer pH 8.0, 0.8% DMSO (with or without the indicated inhibitors) and the proteins indicated in the figure [NEK7 with or without NEK9 (764–976)]. The graph represents the intensity of luminescence reactions made in the absence and presence of different NEK7 inhibitors as indicated in the legend. A3: ATM Kinase Inhibitor; A4: ATM / ATR Kinase Inhibitor; A9: Aminopurvanalol A; D8: GSK-3B Inhibitor VIII; E3: GSK-3 Inhibitor XIII. (**) Indicates *P* < 0·05 calculated using one-Way ANOVA in comparison with column 3 (max. ATP consumption), Multiple comparison test (GraphPad Prism)

In Fig. 3a we can observe a significant decrease in the emitted bioluminescence intensity when we added three different substrate proteins to the NEK7 kinase in the reaction (columns 2–4), relative to the condition without the presence of protein substrate (column 1). This is a consequence of the ATP consumption by NEK7, due to its phosphorylation of substrate proteins/domains: NEK9-regulatory domain(764–976), Mat1(Full- length protein) and CC2D1A(501–940).

After establishing the assays functionality, the next step was to test previously identified inhibitors of NEK7 kinase activity [14], including Aminopurvanalol A (A9), GSK-3b inhibitor VIII (D8) and GSK-3 inhibitor XIII (E3) as well as new inhibitors, not previously tested for NEK7, such as a ATM kinase inhibitor (A3) and a ATM/ATR kinase inhibitor (A4). The reactions were performed as in the previous assay, with the addition of protein NEK9(764–976) as a confirmed protein substrate of NEK7 [13], and 100 μM of each of the different inhibitors.

As shown in Fig. 3b we can see an increase in luminescence intensity in the presence of inhibitors (columns 5–8) relative to the condition with the absence of inhibitor (column 3). These results show that it was possible to optimize the activity assays using luciferase as competitor and that this might be a new method of quantifying kinase activity with different substrates and for performing screening tests for inhibititors.

Human NEKs are a conserved protein kinase family related to cell cycle progression and cell division and are considered potential drug targets for the treatment of cancer and other pathologies. Our new methodology can be very useful for finding promising general and specific candidate inhibitors for any kind of kinase, which then may function as scaffolds to design more potent and selective inhibitors for the treatment of different diseases.

## Discussion

Kinases have major roles in virtually all aspects of the regulation of the cell cycle, signaling pathways and metabolism. Because of that, many diseases, including cancer, result from the deregulation of kinases activity [15–26]. Many efforts are directed towards finding new drugs that modulate kinases in order to treat several diseases.

However, methods to analyze the efficacy of these compounds are quite expensive, complicated or little sensitive. Here, we developed a new bioluminescent method, based on a ATP-dependent red light emitting luciferase. It serves to test the activity of kinases and to identify new kinase inhibitors, by quantifying kinase activity in the presence of different substrates and inhibitors.

Bioluminescence has been extensively used as a reporter to study many processes in molecular biology (e.g.; protein-protein interaction, gene expression, metabolite determination and live cell imaging) [4]. Because the bioluminescence reaction of beetle luciferases depends on the ATP concentration, one of the most useful applications of this technique is to quantify ATP in samples.

Here, we used this approach to verify the activity of kinase proteins. Red emitting-luciferase from *P. hirtus* was previously cloned, purified and characterized [6, 7] and used here to test this concept. In principle, any luciferase could be used for such purpose. Even though the red emitting luciferase is less efficient, this luciferase may be useful for assays containing phenol red derived from cell culture medium, that absorb light in spectral regions emitted by the majority of green/yellow light luciferases.

Furthermore, due to its low K_M_ values for ATP and mainly for luciferin, it would sensibly reduce the cost of the assays. Finally, although the luminometers with photomultipliers are less sensitive to red light, nowadays they are sensitive enough to detect the bright signal of this red-emitting luciferase. CCD camera photo detecting systems have a more uniform spectral response for such purposes.

To test our new method we used creatine kinase and NEK7 kinase protein. Creatine kinase is an important enzyme in the cells energy buffering system. This enzyme has four isoforms, two mitochondrial (ubiquitous and sarcomeric creatine kinase) and two cytosolic (brain and muscle creatine kinase). In mitochondria creatine kinase can convert ATP and creatine, generating phospho-creatine and ADP. In energy demanding cellular sub-sites it can re-convert phospho-creatine in creatine and re-generate ATP. This mechanism is quite important in high energy demanding cells such as working muscle cells or quickly growing cancer cells. In many ovarian cancer cells a CKB over-expression was detected, suggesting an important role in cancer progression. When this same isoform was knocked-down, a decrease in cytosolic glycolysis was observed, which resulted in a tumor suppressive metabolic state [22].

In breast cancer the over-expression of mitochondrial creatine kinase leads to tumor growth by the inhibition of apoptosis [23]. Together, these results indicate that creatine kinase can be a potential target for new drugs. In Fig. 2 we observed that ATP was consumed only in the presence of creatine kinase and creatine, for all four isoforms. With these results we validated our method to test kinases that phosphorylates metabolites, such as all four creatine kinases.

We also tested our method using the serine/threonine protein kinase NEK7. This protein is a member of the “Never in mitosis-gene A” (NIMA)-related kinase (Nek) family and is an important cell cycle regulator. In absence of NEK7 there was a prometaphase arrest with defects in the mitotic spindle, whereas its over-expression results in multi-nucleated cells and a high proportion of apoptotic cells [25]. Additional studies found higher NEK7 expression levels in larynx, breast, colon/rectum and gallbladder cancer [26].

Due to the importance of this protein and the ability to phosphorylate other identified interacting proteins [13] we evaluated the in vitro activity of NEK7. Using our luciferase method we were able to confirm that NEK9, Mat1 and CC2D1A were substrates for NEK7 [13] (Fig. 3a). Afterwards, we also tested if this method is suitable to search for new drugs. After incubating NEK7 with NEK9 (a confirmed bona fide in vivo interactor and substrate of NEK7) and several inhibitors, we verified that the luminescence is higher in the presence of inhibitors in comparison with the absence of inhibitors. This indicates that NEK7 catalytic activity was being inhibited. We conclude that this method can be used to test and screen for kinases inhibitors and may be employed to perform high performance drug testing.

## Conclusion

In this work we developed and validated a luminescence method to quantify kinase activity, which works with different kinases, substrates and even for inhibitor screening. Moreover, we improve the reaction system to be fast, easy and economic, thereby becoming suitable for drug high throughput screening.

## Additional file

Additional file 1: Figure S1. Lack of ATPase contamination in luciferase purification analysis. Demonstrates that the used luciferase preparation is not contaminated with ATP consuming enzymes derived from *E.coli.* **Figure S2.** Purification of proteins used in the assays. Shows purity of the protein samples (luciferase, creatin kinase, NEK7 and 9, Mat1 and CC2D1A) used in the experiments of the paper. (DOCX 252 kb)

## Abbreviations

CC2D1A: Coiled-Coil and C2 Domain Containing 1A
CKB: Brain creatine kinase
CKM: Muscle creatine kinase
Cr: **C**reatine
Mat1: Menage à trois 1
NEK7: NIMA (never in mitosis gene a)-related kinase 7
NEK9: (never in mitosis gene a)-related kinase 9
sMtCK: Sarcomeric mitochondrial creatine kinase
uMtCK: Ubiquitous mitochondrial creatine kinase
