# Supplementary material for: Use of a special Brazilian red-light emitting railroad worm Luciferase in bioassays of NEK7 protein Kinase and Creatine Kinase
Source: BMC Biochem. 2017 Jul 19;18:12. doi: 10.1186/s12858-017-0087-z (PMC5518096; doi:10.1186/s12858-017-0087-z)
Supplement: Additional file 1: Figure S1. — Lack of ATPase contamination in luciferase purification analysis. Demonstrates that the used luciferase preparation is not contaminated with ATP consuming enzymes derived from E.coli. Figure S2. Purification of proteins used in the assays. Shows purity of the protein samples (luciferase, creatin kinase, NEK7 and 9, Mat1 and CC2D1A) used in the experiments of the paper. (DOCX 252 kb) [file 12858_2017_87_MOESM1_ESM.docx]

Supplementary figures

Use of a special Brazilian Red-Light Emitting Railroad Worm Luciferase in bioassays of Nek7 Protein Kinase and Creatine Kinase

Arina Perez, Bruno Aquino, Vadim Viviani and Jörg Kobarg


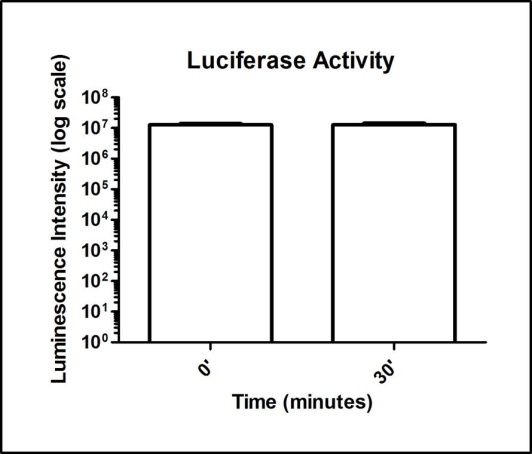


**Supplementary Figure S1. Lack of ATPase contamination in luciferase purification analysis.** Luciferase contamination with ATP consuming enzymes (that could have been co-puryfied from the E.coli lysate) were assessed incubating the purified luciferase with 10uM of ATP for 30 minutes. After incubation, 0,5mM luciferin was added and luminescence was measured. Standard deviation represents five independent experiments.


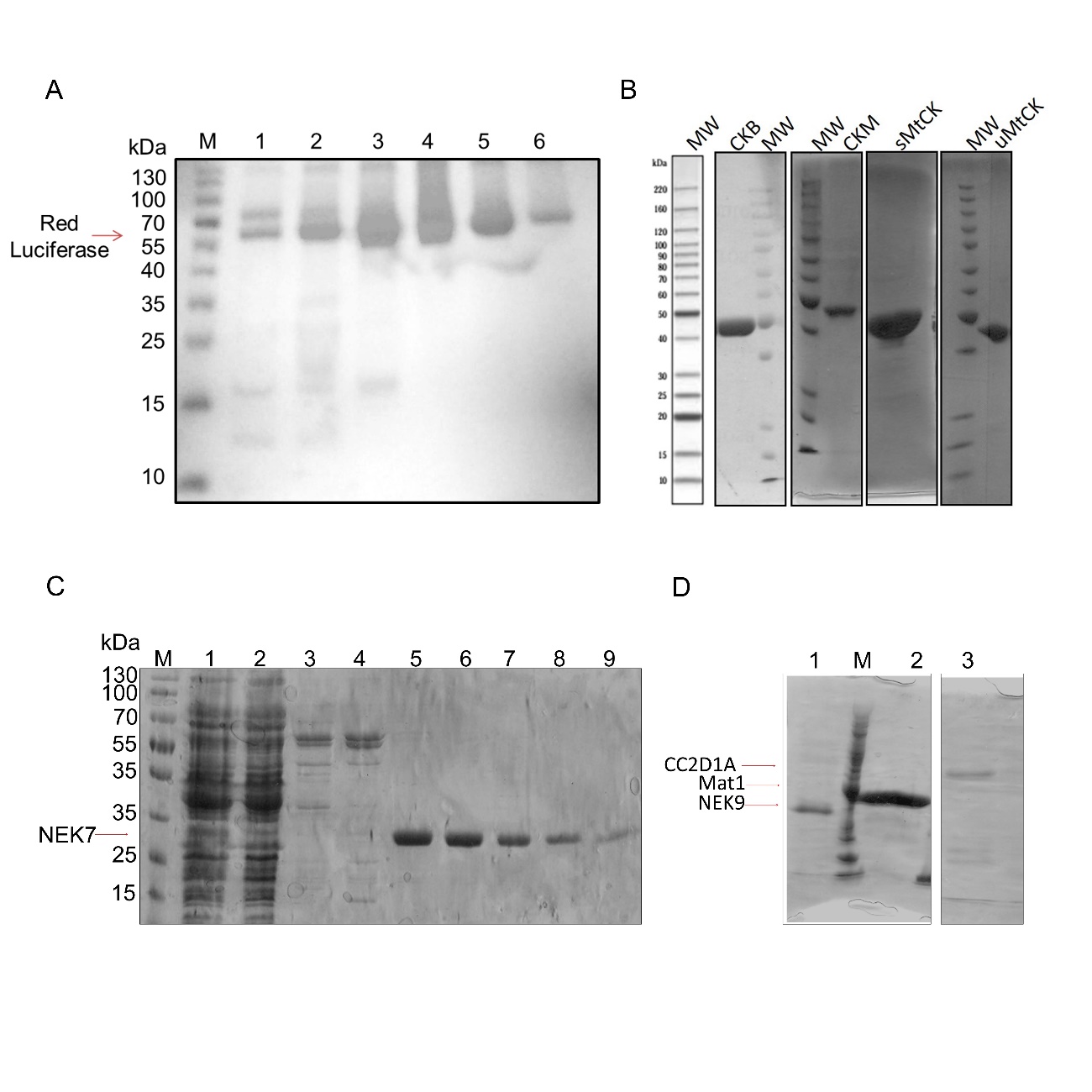


**Supplementary Figure S2 : Purification of proteins used in the assays:**

A) Luciferase purification. SDS-PAGE of affinity chromatography eluates containing 60 kDa 6xHis-Red Luciferase protein.

B) CKB, CKM, sMtCK and uMtCK purification.

C) Nek7 purification. SDS-PAGE of affinity chromatography eluates containing 34 kDa6xHis-NEK7 protein.

D) NEK9 (764-976), Mat1(Full-length), CC2D1A (501-940) purification.
